# Chondroprotective Effects of 4,5-Dicaffeoylquinic Acid in Osteoarthritis through NF-κB Signaling Inhibition

**DOI:** 10.3390/antiox11030487

**Published:** 2022-02-28

**Authors:** Goeun Jang, Seul Ah Lee, Joon Ho Hong, Bo-Ram Park, Do Kyung Kim, Chun Sung Kim

**Affiliations:** 1 Department of Oral Biochemistry, College of Dentistry, Chosun University, Gwangju 61452, Korea; goeun2748@gmail.com (G.J.); seulah21@naver.com (S.A.L.); 2 Nano Bio Research Center, Jeonnam Bioindustry Foundation, Wando 59108, Korea; jhhong7912@jbf.kr; 3 Department of Dental Hygiene, College of Health and Welfare, Kyungwoon University, Gumi 39160, Korea; br8288@naver.com; 4 Oral Biology Research Institute, College of Dentistry, Chosun University, Gwangju 61452, Korea; kdk@chosun.ac.kr

**Keywords:** osteoarthritis, 4,5-dicaffeoylquinic acid, chondroprotective effect, interleukin-1β, articular cartilage

## Abstract

Osteoarthritis (OA) is characterized by cartilage degradation, inflammation, and pain. The dicaffeoylquinic acid (diCQA) isomer, 4,5-diCQA, exhibits antioxidant activity and various other health-promoting benefits, but its chondroprotective effects have yet to be elucidated. In this study, we aimed to investigate the chondroprotective effects of 4,5-diCQA on OA both in vitro and in vivo. Primary rat chondrocytes were pre-treated with 4,5-diCQA for 1 h before stimulation with interleukin (IL)-1β (5 ng/mL). The accumulation of nitrite, PGE_2_, and aggrecan was observed using the Griess reagent and ELISA. The protein levels of iNOS, COX-2, MMP-3, MMP-13, ADMATS-4, MAPKs, and the NF-κB p65 subunit were measured by Western blotting. In vivo, the effects of 4,5-diCQA were evaluated for 2 weeks in a destabilization of the medial meniscus (DMM)-surgery-induced OA rat model. 4,5-diCQA significantly inhibited IL-1β-induced expression of nitrite, iNOS, PGE_2_, COX-2, MMP-3, MMP-13, and ADAMTS-4. 4,5-diCQA also decreased the IL-1β-induced degradation of aggrecan. It also suppressed the IL-1β-induced phosphorylation of MAPKs and translocation of the NF-κB p65 subunit to the nucleus. These findings indicate that 4,5-diCQA inhibits DMM-surgery-induced cartilage destruction and proteoglycan loss in vivo. 4,5-diCQA may be a potential therapeutic agent for the alleviation of OA progression. In this study, diclofenac was set to be administered once every two days, but it showed an effect on OA. These results may be used as basic data to suggest a new dosing method for diclofenac.

## 1. Introduction

Osteoarthritis (OA) occurs due to cartilage wear associated with the long-term use of joints and is present in people over the age of 60 years [1]. OA is characterized by subchondral bone remodeling and osteophytes, as cartilage is damaged by abrasion and inflammation, ultimately interfering with the overall quality of life [2]. From a molecular biology perspective, OA is caused by an increase in extracellular matrix (ECM) degradation in chondrocytes by cartilage-destroying factors such as oxidative stress and the overexpression of inflammatory mediators. Among them, interleukin 1 beta (IL-1β) and tumor necrosis factor-alpha (TNF-α) are the main factors that accelerate degenerative arthritis by inducing the expression of other cartilage ECM-degrading factors (iNOS, PGE_2_, MMPs, and ADAMTS-4) [3].

In particular, inflammation-induced matrix metalloproteinases (MMPs) and ADAMTS-4 degrade proteoglycans (aggrecans), which are the main components of cartilage ECM, interfering with the normal function of the cartilage. As inflammation inducers, they influence the inflammatory environment in synovial membranes and accelerate the expression of cartilage ECM-degrading factors [4]. Therefore, suppression of their expression is considered to be an important factor in the alleviation of OA, and various treatments are focused on improving the symptoms of OA through the suppression of their expression.

As OA is caused by degenerative changes and is incurable, current treatments aim to slow the rate of the disease, control pain, and restore joint function [5]. Anti-inflammatory drugs such as acetaminophen and non-steroidal anti-inflammatory drugs (NSAIDs) are prescribed for first-line drug treatment, but their use is limited in patients with cardiovascular disease because of the high risk of gastrointestinal disturbances or cardiovascular side effects when taken for a long time [5,6,7]. In addition, there are other treatments that suppress inflammation and pain by injecting hyaluronic acid and steroids into the joint cavity, but there is the possibility that pain will worsen, or bacterial infection will occur after administration [8,9]. Recently, platelet-rich plasma therapy, which concentrates plasma isolated from the patient’s own blood and injects it into the treatment site, has been spotlighted, and the treatment rate is currently being discussed positively in many review papers [10,11,12]. Therapeutics are evolving innovatively, but there is still no complete cure. As a non-pharmacological treatment, cartilage replacement surgery is the last resort, and the socio-economic burden that follows is significant. Therefore, there is increasing interest in exercise or joint health functional foods that delay the onset of osteoarthritis by maintaining healthy joints: Glucosamine and MSM are representative joint health functional food [8,13]. However, the benefits of functional foods in improving OA are questionable [13].

The solution to this problem can be found in traditional medicinal plants with significantly fewer side effects and superior pharmacological effects compared to synthetic preparations [14,15]. Traditional medicinal plants have long been used to treat various diseases [16] due to their proven excellent pharmacological effects [17]. In our previous study, we demonstrated the stability and mitigating effects of the leaves of *Anthriscus sylvestris*, a traditional medicinal plant, in OA. The leaves are a strong candidate for development as a functional food [18,19]. However, the seven active ingredients, luteolin-7-O-glucoside (cynaroside), chlorogenic acid (3-CQA), crypto-chlorogenic acid (4-CQA), 3,4-di-caffeoylquinic acid (3,4-diCQA), 3,5-di-caffeoylquinic acid (3,5-diCQA), 4,5-di-caffeoylquinic acid (4,5-diCQA), and luteolin-7-O-(6″malonylglucoside), contained in the hydrothermal extract of Anthriscus sylvestris leaves, have not been sufficiently examined with respect to the alleviation of OA, especially di-caffeoylquinic acid [20,21].

Phytochemicals, such as polyphenols and flavonoids, which are present in high amounts in plant-derived drugs, are known to be effective in alleviating various diseases. Among them, diCQA contains a molecule of caffeic acid and quinic acid connected by an ester bond [21], and is abundant in coffee beans, fruits, and vegetables, including *Gynura divaricata* leaves [22,23]. DiCQA has numerous isomers depending on the position of the double bond, and these influence the cell absorption rates and efficacies. diCQA has anti-inflammatory [24,25], anti-hepatotoxic [26], and neuroprotective effects [27]; it also protects human keratinocytes from oxidative damage [28]. 3,4-diCQA, 3,5-diCQA, and 4,5-diCQA (diCQAs) are present in the hydrothermal extract of A. sylvestris leaves. However, despite these effects, there are no reports on the protective effect of diCQAs on chondrocyte protection for the alleviation of OA. Therefore, in this study, we investigated whether 4,5-diCQA has a protective effect on chondrocytes for the mitigation of OA using in vitro and in vivo experiments.

## 2. Materials and Methods

### 2.1. Reagents

4,5-di-O-caffeoylquinic acid (solubility: 5 mg/mL in DMSO, ≥85% (LC/MS-ELSD), product number: SMB00221), Carrageenan, sulfanilamide, N-(1-naphthyl) ethylenediamine dihydrochloride, 3-(4,5-dimethylthiazol-2-yl)-2,5-diphenyltetrazolium bromide (MTT), and phosphoric acid were purchased from Sigma-Aldrich (St. Louis, MO, USA). The ACAN ELISA kit was purchased from MyBioSource (San Diego, CA, USA), whereas the prostaglandin E2 (PGE_2_) ELISA kit was purchased from R&D Systems (Minneapolis, MN, USA). IL-1β was purchased from ProSpec Protein Specialists (Rehovot, Israel). Collagenase type 2 was purchased from Worthington Biochemical Corporation (Lakewood, NJ, USA). Dulbecco’s modified Eagle’s medium/Nutrient mixture F-12 (DMEM/F12) and penicillin–streptomycin solution were purchased from WelGene (Daegu, Korea). Fetal bovine serum (FBS) was purchased from ATLAS Biologicals (Fort Collins, CO, USA). Primary and secondary antibodies for MMP-13, ADAMTS-4, and iNOS (Abcam, Cambridge, MA, USA); anti-α-tubulin (Thermo Fisher Scientific, Waltham, MA, USA); MMP-1 (Lifespan Biosciences, Seattle, WA, USA), MMP-3, COX-2, and TNF-α (Cell Signaling Technology, Danvers, MA, USA); p-IκB-α, IκB-α, and NF-κB (Invitrogen, Carlsbad, Germany); and PCNA (Santa Cruz Biotechnology, Inc., Dallas, TX, USA) were also purchased.

### 2.2. Cell Culture

In order to isolate chondrocytes, we used a slightly modified method by Kim et al. (2013) [29]. In brief, five-day-old Sprague-Dawley rats were purchased from Damool Science (Daejeon, Korea) for rat primary chondrocyte isolation, and articular cartilage was digested using 0.3% (*w*/*v*) collagenase type II dissolved in DMEM/F12 at 37 °C overnight. All animal management and procedures were approved by the Chosun University Institutional Animal Care and Use Committee (CIACUC2021-S0013). The cells and debris were filtered through a cell strainer (0.45 µm). Approximately 4.5 million chondrocytes were collected from eleven rats killed at the same time. Chondrocytes were seeded at 2 × 10^6^ cells/mL into 6-well cell culture plates with DMEM/F12 containing 10% FBS and 1% penicillin/streptomycin in a humidified incubator with 5% CO_2_ at 37 °C. The chondrocytes were cultured up to 90% confluency and were not passaged during the experiment.

### 2.3. Cell Viability

Viability analysis of 4,5-diCQA on chondrocytes was performed using the MTT assay following the Sigma-Aldrich manufacturer’s protocol. The chondrocytes were treated with 4,5-diCQA (10, 20, 40, 100, and 200 µM) for 24 h. Post-incubation, the MTT solution (5 mg/mL) was added to each well (100 µL/well), and the cells were incubated for another 2 h at 37 °C. Next, the cell culture medium, including the MTT solution, was removed, DMSO (1 mL/well) was added to each well, and the absorbance was measured at 565 nm.

### 2.4. Measurement of Nitric Oxide and PGE_2_ Production

The chondrocytes were pretreated with 4,5-diCQA (10, 20, and 40 µM) for 1 h and then stimulated with IL-1β (5 ng/mL) for 24 h without removing 4,5-diCQA. Nitric oxide production was determined by measuring the accumulation of nitrite in the culture medium. In brief, the culture medium (100 µL) was mixed with 100 µL of the Griess reagent (1% sulfanilamide in 5% phosphoric acid and 0.1% α-naphthylamide in H_2_O) and measured at 540 nm using a microplate reader (Epoch BioTek Instruments Inc., Winooski, VT, USA) to evaluate the accumulation. PGE_2_ production was measured using a Parameter™ prostaglandin E2 assay kit, according to the manufacturer’s protocol.

### 2.5. Western Blotting Analysis

Chondrocytes were pretreated with 4,5-diCQA (10, 20, and 40 µM) for 1 h, and then stimulated with IL-1β (5 ng/mL) for 30 min or 24 h without removing 4,5-diCQA. The cells were then washed using 1× phosphate-buffered saline (PBS) and lysed with a PRO-PREP protein extraction solution (iNtRON Biotechnology, Seongnam-si, Korea) to isolate the whole protein for 30 min on ice. In addition, to isolate cytoplasmic and nuclear proteins, NE-PER™ Nuclear and Cytoplasmic Extraction Reagents (Thermo Fisher Scientific, Waltham, MA, USA) were used according to the manufacturer’s instructions. Additionally, articular cartilages were cut from the explant organ using a blade, and the articular cartilage slices were extracted with a PRO-PREP protein extract to harvest the protein. The cartilage pieces were homogenized in lysis buffer, then incubated on ice for 30 min and centrifuged at 14,000× *g* at 4 °C for 15 min. Protein concentrations were determined using a bicinchoninic acid (BCA) protein assay kit (Pierce, Rockford, IL, USA). Equivalent amounts of lysate protein (10 or 20 µg) were separated on 6, 8, 10, or 12% sodium dodecyl sulfate polyacrylamide (SDS) gels, and then transferred to a polyvinylidene difluoride membrane (Bio-Rad Laboratories, Hercules, CA, USA). The transblotted membranes were blocked with 5% bovine serum albumin in Tris-buffered saline containing 0.1% Tween 20 (TBST) at room temperature for 1 h and then incubated with primary antibodies (1:1000) at 4 °C overnight. The membranes were rinsed three times with TBST and incubated with horseradish peroxidase (HRP)-conjugated secondary antibody (1:10,000) at 25 °C for 1 h. The immunoreactive bands were detected by an enhanced chemiluminescence (ECL) kit (Millipore, Bedford, MA, USA) and visualized using a MicroChemi 4.2 imager (DNR Bioimaging Systems, Jerusalem, Israel).

### 2.6. Aggrecan ELISA Analysis

The chondrocytes were pretreated with 4,5-diCQA (10, 20, and 40 µM) for 1 h, and then stimulated with IL-1β (5 ng/mL) for 24 h without removing 4,5-diCQA. The cultured medium was collected, and aggrecan content was assessed using the aggrecan ELISA kits. All assays were performed in duplicate.

### 2.7. Gelatin Zymography

The chondrocytes were pretreated with 4,5-diCQA (10, 20, and 40 µM) for 1 h and cultured with IL-1β (5 ng/mL) for 24 h without removing 4,5-diCQA. Supernatants from the cultured media (30 µL) were mixed with non-reducing sample buffer (250 mM Tris-HCl [pH 6.8], 40% [*v*/*v*] glycerol, 8% [*w*/*v*] SDS, and 0.01% [*w*/*v*] bromophenol blue) and resolved using 8% SDS-PAGE containing gelatin. After electrophoresis, the gels were rinsed with 2.5% (*v*/*v*) Triton X-100 by gentle shaking for 30 min, followed by washing with distilled water (DW) for 15 min, three times. The gels were then incubated with zymogram renaturing buffer (10 mM CaCl_2_, 50 mM Tris-HCl [pH 7.6], 0.15 M NaCl) at 37 °C for 72 h. After renaturation of the cartilage-degrading enzymes, the gels were stained with 0.1% (*w*/*v*) Coomassie brilliant blue R-250 for 30 min at room temperature, destained until clear bands were visible, and imaged using a digital camera (G16, Canon, Tokyo, Japan). The clearance white zone in the band indicates gelatin degradation.

### 2.8. Animals

Male, specific pathogen-free Sprague–Dawley rats (8 weeks old), weighing 300 g, with four in each group for a total of twenty-eight, were purchased from Damool Science (Daejeon, Korea). The animals were nurtured in a controlled environment (temperature: 21 ± 1 °C; humidity: 55 ± 5%; 12-h light/dark cycle) and were allowed free access to commercial feed. All animals were handled by procedures from the National Institutes of Health Guide for the Care and Use of Laboratory Animals [30]. The surgical model of destabilization of the medial meniscus (DMM) was established in Sprague–Dawley rats to induce OA. Prior to the experiment, the rats were acclimatized to the environment for 1 week. Approval to perform the DMM surgery was provided by the Chosun University Institutional Animal Care and Use Committee (CIACUC2021-S0015).

### 2.9. DMM-Induced OA Model in Rats

The rats were divided into seven groups of four rats each: Group 1 (normal), Group 2 (Sham, 0.9% saline), Group 3 (DMM, 0.9% saline), and Group 4–7 (5, 10, and 20 mg/kg 4,5-diCQA and 10 mg/kg diclofenac); each drug was administered as is. DMM surgery was performed by incising the medial meniscotibial ligament (MMTL) to induce OA above the right and left knees [18,31]. For this operation, the rats were anesthetized with 2.5% isoflurane, and then the MMTL was incised. In the Sham group, only the skin was incised and the MMTL was not incised. Two weeks after DMM surgery, the 4,5-diCQA groups were orally administered 4,5-diCQA (5, 10, or 20 mg/kg) and the diclofenac group was orally administered diclofenac (10 mg/kg). 4,5-diCQA was dissolved in DMSO at a high concentration and then diluted with saline to match the concentration. Diclofenac was dissolved in saline. The drugs were orally administered once every 2 days for 2 weeks. The sham and DMM-only groups were orally administered with saline for the same period. Subsequently, all the rats were sacrificed on the same day.

### 2.10. Histology Analysis and Staining

Extracted articular cartilages were fixed in 10% neutral-buffered formalin for one day at 4 °C, and then decalcified with 0.5 M EDTA (pH 7.4) for seven days at 4 °C. Following these steps, the articular cartilage was dehydrated through a series of ethanol solutions and embedded in paraffin blocks. After that, lateral serial sections of 4 µm thickness were sliced and stained with Safranin O/Fast Green. An EVOS Core microscope (Thermo Fisher Scientific, Waltham, MA, USA) was used to digitally photograph the stained sections. The stained sections were scored according to the Osteoarthritis Research Society International (OARSI) advanced Osteoarthritis Cartilage Histopathology Assessment System (0–6.5), and a summed OARSI score was used to analyze the degree of articular cartilage destruction [32].

### 2.11. Statistical Analysis

All data were obtained from independent experiments. The results are expressed as mean ± standard deviation (SD). One-way analysis of variance (ANOVA) from Dunnett’s test was employed for multiple comparisons using GraphPad Prism 5.0 software (GraphPad Software Inc., San Diego, CA, USA). Statistical significance was set at ### *p* < 0.005 compared with the control group and * *p* < 0.5, ** *p* < 0.05, *** *p* < 0.005 compared with the IL-1β-treated group.

## 3. Results

### 3.1. Effect of 4,5-diCQA on the Viability of Rat Primary Chondrocytes

**Figure 1:**
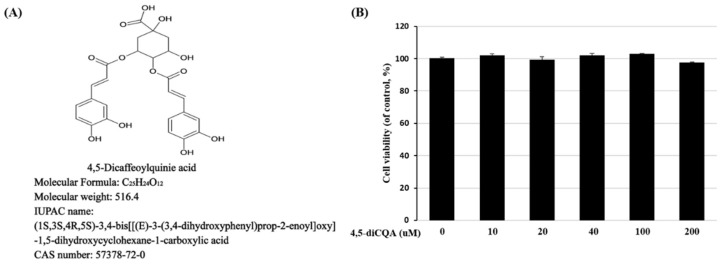
Effects of 4,5−diCQA on rat primary chondrocyte viability. (**A**) Chemical formula of 4,5−diCQA (https://pubchem.ncbi.nlm.nih.gov/compound/4_5-Di-O-caffeoylquinic-acid, accessed on 20 November 2021). (**B**) Cells were treated with 4,5-diCQA (10, 20, 40, 100, and 200 μM) for 24 h, and viability was determined by MTT assay. Cells incubated without 4,5-diCQA were used as controls and were considered 100% viable. Data are represented as mean ± SD of three independent experiments.

Figure 1A represents the chemical formula of 4,5-diCQA. To investigate the toxicity of 4,5-diCQA, rat primary chondrocytes were treated with 10, 20, 40, 100, and 200 µM 4,5-diCQA for 24 h. 4,5-diCQA was non-toxic up to a concentration of 200 µM (Figure 1B).

### 3.2. Effects of 4,5-diCQA on IL-1β-Induced Nitrite and PGE_2_ Expression in Rat Primary Chondrocytes

**Figure 2:**
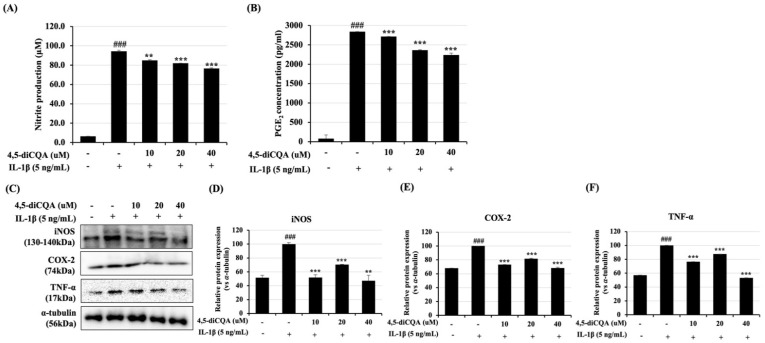
Inhibitory effects of 4,5−diCQA on IL-1β-induced nitrite, PGE_2_, iNOS, COX-2, and TNF-α in rat primary chondrocytes. Cells were pre-treated with 4,5-diCQA (10, 20, and 40 μM) for 1 h, followed by IL-1β (5 ng/mL) stimulation for 24 h. (**A**) Nitrite production was determined in the cultured medium using a Griess reagent. (**B**) PGE_2_ production was determined in the cultured medium using an ELISA kit after 24 h. (**C**) Expression of the iNOS, COX-2, and TNF-α was determined using Western blot analysis. α-Tubulin served as an internal control. (**D**–**F**) Quantitative data of (**C**) were analyzed using the ImageJ bundled with Java 1.8.0_172 software. *n* = 5 per group. Data are represented as mean ± SD of three independent experiments. ### *p* < 0.005 vs. control group; ** *p* < 0.05, and *** *p* < 0.005 compared with the IL-1β-treated group.

Inflammation is the main cause of OA exacerbation. Therefore, the expression levels of nitrite and PGE_2_ were first examined in the supernatant of IL-1β-treated rat primary chondrocytes. First, the rat primary chondrocytes were pre-treated with diCQA (10, 20, and 40 µM) for 1 h, and then treated with IL-1β (5 ng/mL) for 24 h. In the group subjected to only IL-1β treatment, the expression levels of nitrite and PGE_2_ were significantly increased (Figure 2A,B). However, in the group pre-treated with 4,5-diCQA, the expression levels of nitrite and PGE_2_ decreased in a concentration-dependent manner, even when treated with IL-1β. In addition, the levels of inflammatory mediators, such as iNOS, COX-2, and TNF-α, and inflammatory cytokines, were increased only in IL-1β-treated rat primary chondrocytes, but not in the group pre-treated with 4,5-diCQA (Figure 2C). These findings indicate that 4,5-diCQA has potential anti-inflammatory effects by suppressing the IL-1β-induced inflammatory response.

### 3.3. Effects of 4,5-diCQA on IL-1β-Induced Expression of Matrix-Degrading Enzymes in Rat Primary Chondrocytes

**Figure 3:**
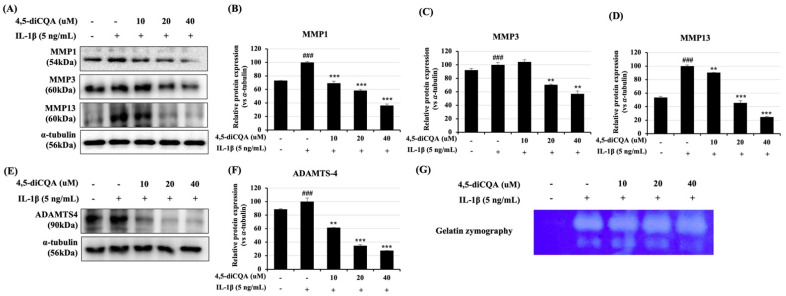
Inhibitory effect of 4,5-diCQA on IL-1β-induced MMP-1, MMP-13, and ADAMTS-4 in rat primary chondrocytes. Cells were pre−treated with 4,5-diCQA (10, 20, and 40 μM) for 1 h, followed by IL-1β (5 ng/mL) stimulation for 24 h. (**A**,**E**) Protein levels of MMP-1, MMP-13, and ADAMTS-4 were determined using Western blot analysis. α-Tubulin served as an internal control. (**B**–**D**) Quantitative data of (**A**) were analyzed using the ImageJ bundled with Java 1.8.0_172 software. (**F**) Quantitative data of (**E**) were analyzed by using the ImageJ bundled with Java 1.8.0_172 software. (**G**) Activity of MMPs was measured in conditioned medium using gelatin zymography. Data are represented as mean ± SD of three independent experiments. *n* = 5 per group. ### *p* < 0.005 vs. control group; ** *p* < 0.05, and *** *p* < 0.005 compared with the IL-1β-treated group.

Inflammatory mediators such as nitric oxide and PGE_2_ promote the secretion of matrix-degrading enzymes such as MMPs and ADAMTS-4. MMPs and ADAMTS-4 are enzymes that degrade aggrecan (ACAN), and ECM degradation is a prominent feature of OA. Therefore, the efficacy of 4,5-diCQA was examined in IL-1β-treated rat primary chondrocytes by evaluating the expression levels of MMP-1, -3, -13, and ADAMTS-4. The expression levels of MMP-1, MMP-3, MMP-13, and ADAMTS-4 increased in the group treated with IL-1β alone. However, the expression levels of these enzymes were significantly reduced in the group that was pre-treated with 4,5-diCQA and with IL-1β (Figure 3A,B). Moreover, MMP expression level increased in the IL-1β-only group during gelatin zymography using the supernatant of the culture medium (Figure 3C). These results indicate that 4,5-diCQA inhibited cartilage-degrading enzymes with IL-1β treatment.

### 3.4. Effects of 4,5-diCQA on IL-1β-Induced ACAN Degradation in Rat Primary Chondrocytes

**Figure 4:**
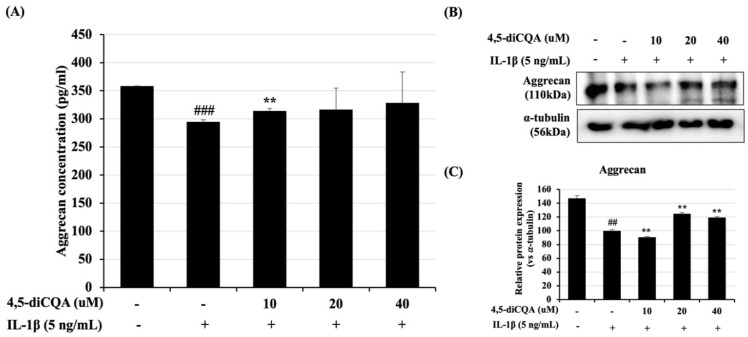
Inhibitory effect of 4,5-diCQA on IL-1β-induced ACAN degradation in rat primary chondrocytes. Cells were pre-treated with 4,5-diCQA (10, 20, and 40 μM) for 1 h, followed by IL-1β (5 ng/mL) stimulation for 24 h. (**A**) Conditioned medium was prepared for ACAN ELISA assay. (**B**) Protein levels of ACAN were determined using Western blot analysis. α-Tubulin served as an internal control. (**C**) Quantitative data of (**B**) were analyzed using the ImageJ bundled with Java 1.8.0_172 software. *n* = 5 per group. ANOVA and Dunnett tests were used to evaluate the significance of the results. Data are represented as mean ± SD of three independent experiments. ## *p* < 0.05 and ### *p* < 0.005 vs. control group; ** *p* < 0.05 compared with IL-1β-treated group.

ACAN is a component of cartilage ECM, but with IL-1β treatment, degradation of ACAN is promoted by inflammation. This experiment was performed to confirm whether 4,5-diCQA prevents the degradation of ACAN in rat primary chondrocytes. The expression level of ACAN was measured by ELISA using the supernatant of the cultured media, and by Western blot using the cell lysates. The expression level of ACAN was decreased in the group treated only with IL-1β in rat primary chondrocytes, but the group that was pre-treated with 4,5-diCQA was significantly increased (Figure 4). These findings suggest that 4,5-diCQA has a potential chondroprotective effect by suppressing the degradation of ACAN in the IL-1β-treated group.

### 3.5. Effects of 4,5-diCQA on the NF-κB Signaling Pathway in IL-1β-Treated Rat Primary Chondrocytes

**Figure 5:**
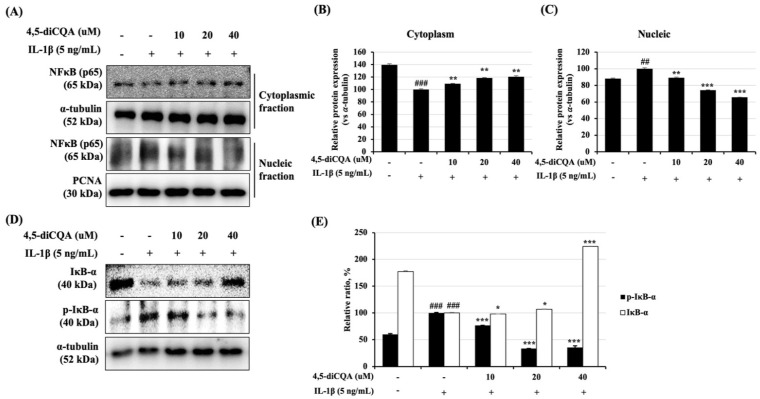
Effects of 4,5-diCQA on IL-1β-induced activation of NF-κB in rat primary chondrocytes. Cells were pre-treated with 4,5-diCQA (10, 20, and 40 μM) for 1 h, followed by IL-1β (5 ng/mL) stimulation for 1 h. (**A**) Phosphorylation levels of IκB-α and NF-κB p65 translocation to the nucleus were determined using Western blot analysis. α-Tubulin and PCNA were used as cytosolic and nuclear internal controls, respectively. (**B**,**C**) Quantitative data of (**A**) were analyzed using ImageJ bundled with Java 1.8.0_172 software. (**E**) Quantitative data of (**D**) were analyzed using the ImageJ bundled with Java 1.8.0_172 software. *n* = 5 per group. Data are represented as mean ± SD of three independent experiments. ## *p* < 0.05 and ### *p* < 0.005 vs. control group; * *p* < 0.5, and ** *p* < 0.05, and *** *p* < 0.005 compared with the IL-1β-treated group.

NF-κB is an important transcription factor that regulates the transcription of cartilage-degrading enzymes, such as MMPs, the ADAMTS family, and inflammatory mediators. Therefore, NF-κB activity in IL-1β-treated rat primary chondrocytes was examined to determine the efficacy of 4,5-diCQA. With 30 min of IL-1β treatment, the NF-κB p65 subunit translocated from the cytoplasm to the nucleus, and the expression level increased (Figure 5A). The phosphorylation and degradation of IκB occurred simultaneously in the cytoplasm. However, in the group that was pre-treated with 4,5-diCQA for 1 h and with IL-1β, phosphorylation and degradation of IκB were suppressed, and the transfer of the NF-κB p65 subunit from the cytoplasm to the nucleus was also suppressed (Figure 5). These findings suggest that the transcription of NF-κB is regulated by the chondroprotective effects of 4,5-diCQA.

### 3.6. Effects of 4,5-diCQA Administration on Macroscopic and Histological Parameters in the Articular Cartilage of the Rat OA Model

**Figure 6:**
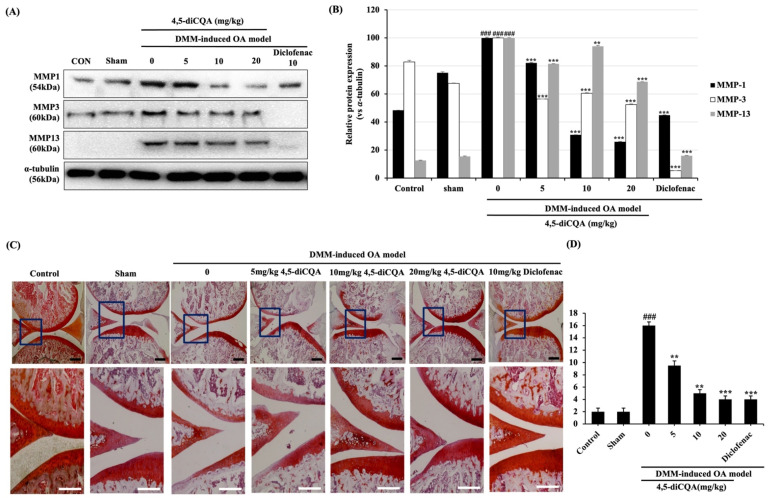
Histological evaluation of cartilage-protective effect of 4,5-diCQA against cartilage degradation in a DMM model. After sham or DMM surgery, rats received gavage of distilled water, 4,5-diCQA (5, 10, and 20 mg/kg bodyweight), or diclofenac sodium (10 mg/kg bodyweight) every other day for 2 weeks. (**A**) Protein levels of MMP-1, -3, and -13 were determined using Western blot analysis. α−Tubulin served as an internal control. (**B**) Quantitative data of (**A**) were analyzed using the ImageJ bundled with Java 1.8.0_172 software. *n* = 5 per group. Data are represented as mean ± SD of three independent experiments. ### *p* < 0.005 compared with sham group; ** *p* < 0.05, and *** *p* < 0.005 compared with the DMM-induced group. (**C**) Histological analysis of cartilage destruction was evaluated by Safranin O/Fast green staining. Black scale bars, 500 μm. White scale bars, 100 μm. (**D**) Osteoarthritis Research Society International (OARSI) advanced Osteoarthritis Cartilage Histopathology Assessment System. ANOVA and Dunnett tests were used to evaluate the significance of the results. ### *p* < 0.005 compared with sham group; ** *p* < 0.05, *** *p* < 0.005 compared with DMM group.

DMM is a widely used surgical technique, in which the medial meniscus is incised; the model has a pathology like that of human OA. The effects of 4,5-diCQA on the DMM-altered OA cartilage structure were determined using Western blot by confirming MMP families and using Safranin O/Fast green staining based on the OARSI score (Table 1) [32]. The expression of MMP-1, -3, and -13 significantly increased in DMM-alone-induced rats, but the expression level was reduced in DMM-induced rats administered orally with 4,5-diCQA. 4,5-diCQA markedly suppressed the protein expression of DMM-induced MMP-1, -3, -13, which coincides with the in vitro results (Figure 6A,B). Cartilage damage was observed only in the OA group, whereas it was not observed in rats subjected to DMM and in those that were orally administered with 4,5-diCQA (Figure 6C). The DMM-induced OA group had an OARSI score of 16 ± 0.57, which indicated cartilage destruction and erosion. However, in the 5, 10, and 20 mg/kg 4,5-diCQA and 10 mg/kg diclofenac-treated OA groups, the OARSI scores were 10 ± 0.76, 5 ± 0.57, 4 ± 0.57, and 4 ± 0.57, respectively, indicating a significant reduction in cartilage destruction (Figure 6D). These results suggest that 4,5-diCQA alleviates OA in vivo.

## 4. Discussion

Osteoarthritis, a debilitating degenerative joint disease found primarily in people over the age of 60 years, is a major cause of disability that increases medical costs and reduces the quality of life [33]. The pathogenic mechanism of OA has not been elucidated to date, but accumulated research indicates that inflammation plays a vital role in the initiation and development of OA [33,34,35]. The imbalance in chondrocyte metabolism due to inflammation causes an overall shift toward catabolism over anabolism by excessively increasing the expression of inflammatory cytokines and substrate-degrading enzymes, eventually leading to apoptosis and cartilage destruction [36,37]. Therefore, research on the mechanism of protecting chondrocytes may be a strategy to delay or improve the development of OA, and plant-derived components with fewer side effects and excellent pharmacological effects are attracting attention as ideal drugs for OA [38,39,40]. In our previous study, A. sylvestris leaf water extract (AELAS) significantly suppressed the expression of IL-1β-induced expression of OA-catabolic factors (nitric oxide, iNOS, COX-2, PGE_2_, MMP-3, -13, and ADAMTS-4) and degradation of ACAN, collagen type II, and proteoglycan in rat primary chondrocytes [17]. In addition, AELAS inhibited DMM-surgery-induced cartilage destruction and proteoglycan loss [17]. Component analysis, to identify the active ingredients of AELAS, confirmed that a large number of CQA-derived ingredients were present. Therefore, this study suggests that 4,5-diCQA exhibits profitable chondroprotective effects by suppressing the expression of pathological factors that influence OA, including the degradation of articular ECM, nitrosative damage, and the expression of proinflammatory cytokines and mediators, via the NF-κB signaling pathways in vitro and in vivo.

IL-1β is a potent catabolic factor involved in the pathogenesis of OA. It induces the expression levels of other OA catabolic factors, such as iNOS, nitric oxide, COX-2, PGE_2_, TNF-α, MMPs, and ADAMTSs, which relate to chondrocyte dysfunction and ultimately accelerate the initiation and progression of ECM degradation in chondrocytes [35]. In particular, nitric oxide and PGE_2_, which are highly expressed in OA patients, are the early mediators of inflammation and inhibit the synthesis of collagen type II by inducing the expression of other catabolic factors [41,42]. Therefore, the inhibition of IL-1β-induced inflammatory mediators (iNOS, nitric oxide, COX-2, and PGE_2_) can alleviate OA pathogenesis and reduce pain, inflammation, and proteoglycan loss [35,43]. In this study, the expression of nitric oxide, PGE_2_, iNOS, and COX-2 improved upon IL-1β treatment. However, pre-treatment with 4,5-diCQA alleviated this effect. These results are related to those of previous studies that ameliorated the effects of OA; Liu et al. reported that treatment with the CQA-rich fraction of Periploca forrestii (CQAF) significantly blocked IL-1β-induced expression of nitric oxide, PGE_2_, COX-2, and iNOS in MH7A cells (human rheumatoid arthritis synovial cell line) [44]. MMPs are a family of proteinases that contribute to the degradation of collagen type II and proteoglycan, and elevated expression of MMP-13 is characteristic of OA chondrocytes [45,46]. ADAMTS is also deeply involved in OA pathogenesis, and ADAMTS-4 is considered the primary aggrecanase [44]. In this study, we noted that IL-1β treatment increased the expression and activity of MMP-1, MMP-3, MMP-13, and ADAMTS-4. In addition, IL-1β treatment induced the degradation of ACAN. However, pretreatment with 4,5-diCQA inhibited the degradation of ACAN by suppressing the IL-1β-induced activity of MMPs (1, 3, and 13) and ADAMTS-4. Similar effects of 4,5-diCQA were observed in previous studies on the anti-arthritic effects of natural products. Tran et al. reported that avenanthramide-C (Avn-C) isolated from oats suppressed IL-1β-induced expression of MMP-3, -12, and -13 in mouse articular chondrocytes, and Feng et al. reported that oleuropein inhibited the IL-1β-induced expression of inflammatory mediators (nitrite oxide, PGE_2_, COX-2, and iNOS) and ECM proteinases (MMP-1, MMP-3, MMP-13, and ADAMTS-5) [23,43]. These results clearly suggest that 4,5-diCQA has a chondroprotective effect against IL-1β-related induction and the development of OA.

The MAPK and NF-κB pathways are critical in certain chronic inflammatory diseases, such as OA [17,47,48,49]. The MAPK family includes various extracellular signal-regulated kinases, of which ERK modulates chondrocyte proliferation and gene expression; p38 and JNK affect the inflammation and destruction of articular cartilage [50]. Phosphorylated MAPKs (p-ERK1/2, p-JNK, and p-p38) relate MMPs expression and cartilage degradation [50]. The NF-κB pathway is modulated by the MAPKs phosphorylation and is included in the regulation of inflammatory mediators as well as OA progression [51,52]. Normally, NF-κB is localized to the cytoplasm with its inhibitor subunit IκB-α, but IL-1β induced phosphorylation and degradation of IκB, and results in the translocation of NF-κB p65 subunit into the nucleus, resulting in the induction of inflammatory mediators [51,53,54]. Therefore, repression of these pathways is crucial in suppressing inflammation, and several studies have reported that certain plants and naturally derived compounds, such as Caragana sinica root extract, Punica granatum extract, oleuropein, and CQAF, show anti-arthritic activity by regulating the MAPK and NF-κB pathways [34,51,52,55]. In this study, 4,5-diCQA treatment inhibited the IL-1β-induced phosphorylation of MAPKs (ERK1/2, JNK, and p38), the degradation of IκB-α, and the translocation of the NF-κB p65 subunit. These results suggest that the chondroprotective effect of 4,5-diCQA may be mediated by MAPKs and the NF-κB signaling pathway. 

In vivo, we established a rat OA model through surgical destabilization of the medial meniscus (DMM) to evaluate the protective effect of 4,5-diCQA on cartilage degradation. The DMM-induced OA model is a meniscus dissection and degeneration induced, and it is widely used to evaluate the efficacy of the drug because it resembles the development of osteoarthritis associated with human aging [56]. Furthermore, histological staining provides specific information about the pathological conditions of articular cartilage, such as changes in chondrocytes and matrix components, and is, therefore, commonly used to evaluate the improvement of arthritis [57]. As a result of oral administration of 4,5-diCQA once every 2 days for 2 weeks, the severity of cartilage degradation in the DMM-induced OA model was alleviated by suppressing catabolic activity and reducing damage in chondrocytes, and this result was consistent with the OARSI score. The use of diclofenac as a positive control was two or three times a day, but it was shown to be effective for OA even when administered once every two days, which can be presented as basic data for a new dosing regimen of diclofenac. Therefore, the progressive efficacy of diclofenac for OA can be expected through additional pharmacological experiments in the future.

## 5. Conclusions

In conclusion, pre-treatment with 4,5-diCQA effectively inhibits the expression of IL-1β-induced inflammatory factors (nitrite oxide, PGE_2_, iNOS, and COX-2) and cartilage-degrading enzymes (MMP-1, -3 -13, and ADAMTS-4). Furthermore, 4,5-diCQA also protects the ACAN, which is a component of the chondrocyte ECM, from degradation due to IL-1β treatment and DMM surgery. These results suggest that 4,5-diCQA is the active ingredient of AELAS, with OA-ameliorating and chondroprotective effects. Like 4,5-diCQA, it is considered that diclofenac may be effective for OA even when taken at a dose of 10 mg/kg by rats once every 2 days for 2 weeks.

## Figures and Tables

**Table 1:** Osteoarthritis Research Society International (OARSI) score.

Grade	Osteoarthritic Damage
Grade 0	Normal cartilage
Grade 1	Threshold in cartilage for OA without loss of cartilage
Grade 2	Discontinuity of the superficial layer
Grade 3	Erosion of the matrix cracks by extending cartilage to <25% of the articular surface
Grade 4	Erosion of the matrix cracks by extending cartilage to <25–50% of the articular surface
Grade 5	Erosion of the matrix cracks by extending cartilage to <50–75% of the articular surface
Grade 6	Erosion of the matrix cracks by extending cartilage to <75% of the articular surface

## Data Availability

Data is contained within the article.
